# Reciprocal regulation between taurine and glutamate response via Ca^2+^- dependent pathways in retinal third-order neurons

**DOI:** 10.1186/1423-0127-17-S1-S5

**Published:** 2010-08-24

**Authors:** Simon Bulley, Wen Shen

**Affiliations:** 1 College of Biomedical Science, Florida Atlantic University, Boca Raton, FL 33431, USA

## Abstract

Although taurine and glutamate are the most abundant amino acids conducting neural signals in the central nervous system, the communication between these two neurotransmitters is largely unknown. This study explores the interaction of taurine and glutamate in the retinal third-order neurons. Using specific antibodies, both taurine and taurine transporters were localized in photoreceptors and Off-bipolar cells, glutamatergic neurons in retinas. It is possible that Off-bipolar cells release juxtaposed glutamate and taurine to activate the third-order neurons in retina. The interaction of taurine and glutamate was studied in acutely dissociated third-order neurons in whole-cell patch-clamp recording and Ca^2+^ imaging. We find that taurine effectively reduces glutamate-induced Ca^2+^ influx via ionotropic glutamate receptors and voltage-dependent Ca^2+^ channels in the neurons, and the effect of taurine was selectively inhibited by strychnine and picrotoxin, but not GABA receptor antagonists, although GABA receptors are present in the neurons. A CaMKII inhibitor partially reversed the effect of taurine, suggesting that a Ca^2+^/calmodulin-dependent pathway is involved in taurine regulation. On the other hand, a rapid influx of Ca^2+^ through ionotropic glutamate receptors could inhibit the amplitude and kinetics of taurine-elicited currents in the third-order neurons, which could be controlled with intracellular application of BAPTA a fast Ca^2+^ chelator. This study indicates that taurine is a potential neuromodulator in glutamate transmission. The reciprocal inhibition between taurine and glutamate in the postsynaptic neurons contributes to computation of visual signals in the retinal neurons.

## Introduction

Taurine is a sulfur containing amino acid structurally similar to the neurotransmitters glycine and GABA (Gamma aminobutyric acid). It is the most abundant free amino acid in retina and the second most abundant free amino acid in the central brain after glutamate [1]. Although taurine has been found to play a large role in neural development, osmoregulation and neural protection, the function of taurine in neurotransmission and modulation remains poorly understood. In many studies taurine has been considered as a low affinity ligand binding to glycine or GABA receptors [2,3]. Yet, studies also indicate that taurine-produced effects can not be simply repeated by either glycine or GABA [4-6]. The absence of any molecular evidence of a specific receptor and a lack of a specific antagonist for taurine make it difficult to differentiate its effects from glycine and GABA. Taurine is known to have its own transporters expressed in both neurons and astroglial cells [7] and in many cases, taurine transporters are found in glutamatergic neurons, suggesting that taurine and glutamate may be released from the same neurons. This feature in general is distinct from glycine and GABA that are released from the neurons other than glutamatergic cells.

Glutamate is the major excitatory neurotransmitter conducting visual signals within retina. In general glutamate releases from presynaptic neurons and transmits signals upon activation of metabotropic and ionotropic receptors in the postsynaptic neurons. Activation of metabotropic receptors usually triggers intracellular transduction pathways associated with changes of [Ca^2+^]_i_ levels, leading to a large amplification of glutamate signals; whereas activation of ionotropic receptors directly changes the cell membrane potential by cation influx. Some ionotropic glutamate receptors are Ca^2+^ permeable. Ca^2+^ entrance triggers intracellular second-messenger pathways that lead to alterations in cellular and molecular levels in neurons. Therefore regulation of Ca^2+^ permeable glutamate receptors in neurons can exert a large influence in neuronal signals. This study is to examine the effect of taurine on regulation of Ca^2+^ permeable ionotropic glutamate receptors in the retinal neurons.

In retinas, taurine is primarily found in the glutamatergic neurons, photoreceptors and bipolar cells of rat [8], goldfish [9-11] and Cynomologous monkey [12]. Taurine uptake has been also observed in amacrine and ganglion cells, as well as non-neurons, Müller cell and pigmentary epithelium cells, in the early developmental and young ages of animals [13-16]. Since amacrine and ganglion cells receive glutamate inputs from bipolar cells, as well as glycine and GABA inputs from surrounding amacrine cells if taurine is released from bipolar cells, it would juxtapose with these neurotransmitters on amacrine and ganglion cells. Because ganglion cells convey retinal neural signals to the brain via optic nerves, the balance between the excitatory and inhibitory signals in the neurons is critical for visual signal processing from retina to the central brain. The effect of taurine in regulation of glutamate signals in ganglion cells has not yet been determined.

We used amphibian retinal amacrine and ganglion cells, the third-order neurons, as a model system to study the interactions of taurine and glutamate signals. To avoid retinal network effects, isolated neurons were used in this study. We characterized taurinergic neurons using specific antibodies against taurine and the taurine transporter. The results indicate that photoreceptors and Off-type bipolar cells are taurinergic neurons in the retina. We find that the effect of taurine on the third-order neurons suppresses Ca^2+^ influx through both ionotropic glutamate receptors and voltage-gated Ca^2+^ channels. Taurine regulation of ionotropic glutamate receptors was dose-dependent and via a Ca^2+^ sensitive calmodulin kinase II (CaMKII) intracellular pathway and the contribution of protein kinase A (PKA) to a lesser extent. On the other hand, taurine-elicited responses were effectively reduced by rapid [Ca^2+^]_i_ increase via AMPA and kainate receptors in the third-order neurons. The effects of taurine and glutamate reciprocally inhibit each other, linked by [Ca^2+^]_i_. Our study indicates that taurine is important for control of glutamate-induced Ca^2+^ overload in the retinal third-order neurons.

## Materials and methods

### Cell dissociation

Larval tiger salamanders (*Ambystoma tigrinum*), were purchased from Kons Scientific (Germantown, WI) and Charles Sullivan (Nashville, TN). The animals were kept in aquaria at a constant 12^o^C under a 12-hour dark-light cycle with continuous filtration. The retinas were collected from animals that had been kept at least six hours in the dark. Briefly, the animals were decapitated, double-pithed and the eyes were enucleated. All procedures were performed in accordance with the guidelines of National Institutes of Health Guide for the Care and Use of Laboratory Animals and approved by the University's Animal Care Committee.

The retinal tissue was removed from the eyecup in Ringer’s solution consisting of (in mM):111 NaCl, 2.5 KCl, 1.8 CaCl_2_, 1 MgCl_2_, 5 HEPES and 10 Dextrose, adjusted to pH = 7.7. Retinal tissue was then dissociated in freshly prepared enzymatic tissue dissociation solution containing 50μl papain (12 Uml^-1^), 400μl of Ringer’s solution containing (in mM) 5 L-Cysteine and 1 EDTA (adjusted to pH 7.4) for 20-35 minutes at room temperature. The enzymatically treated retina was washed before being mechanically dissociated, by gentle shaking, in Ringer’s solution. The dissociated cells were seeded on 18mm glass cover slips freshly coated with lectin and allowed to set for 20 minutes before use. The cover slip was then placed in the recording chamber containing Ringer solution on an Olympus BX51WI microscope equipped with a CCD camera linked to a monitor. All experimentation was done at room temperature and within a few hours of preparation.

### Electrophysiological recording system

Whole-cell recordings were performed on amacrine and ganglion cells using an EPC-10 amplifier and HEKA Patchmaster software. These third-order neurons were identified based on their distinctive large transient sodium currents for whole cell recording. Low resistance (5-10MΩ) electrodes were pulled from borosilicate glass (Drummond Scientific Co) with the P-97 micropipette puller (Sutter Instrument Co). The electrodes were filled with a high-potassium solution containing (in mM): 100 K-gluconate, 1 MgCl_2_, 5 EGTA and 10 HEPES, with an ATP regenerating cocktails consisting of (in mM): 20 ATP, 40 Phosphocreatine and 2 Creatine Phosphokinase (adjusted to pH 7.4). Cell responses were recorded at a holding potential of -60mV.

The isolated cell preparation was constantly perfused with oxygenated Ringer’s solution. A DAD-VM automated superfusion system (ALA Scientific Instruments) was used for local drug application. Glutamate, NMDA, AMPA and kainic acid with or without Taurine pre-perfusion were all applied for 3-second intervals. Any antagonist that was also applied during experimentation was first pre-perfused at a low pressure. All drugs were prepared in stock solutions before being diluted to their final concentrations (listed in results section) in the perfusion solution on the day of use.

### Immunocytochemistry

Freshly enucleated eyeballs were fixed in either 2.25% glutaraldehyde with 2% paraformaldehyde (for anti-taurine labeling) or 4% paraformaldehyde (for anti-taurine transporter labeling) in Ringer’s solution for 10 minutes, before 10 minutes fixation of the eyecups after corneas were removed. The eyecups were then thoroughly washed in the Ringer’s solution. The fixed eyecups were gradually dehydrated in 15%, 20% and then 30% sucrose solutions (in the Ringer’s solution) before being kept overnight in 30% sucrose. The eyecups were embed in the optimal cutting temperature (OCT) compound (Ted Pella, Redding CA), frozen and dissected at 12-20µm thickness. The frozen sections were collected on silane coated slides, dried and stored at -80°C.

Retinal frozen sections were rinsed with PBS and treated with an antibody blocking reagent “cocktail” consisting of 10% goat serum, 1% BSA in 0.3% Triton X-100 with 0.1% Tween in PBS (PBST) for 20 minutes. The retinal sections were then incubated with an affinity purified rabbit anti-taurine polycolonal antibody (Lot: LV1503470, Chemicon), with a 1:200 concentration, or rabbit anti-taurine transporter polyclonal antibody (Lot: LV1390267, Chemicon) with 10µg/mL for 2 hours. After being washed for 35 minutes, the sections were incubated with a secondary antibody, the goat-anti-rabbit Cy-3-conjugated (1:600) antibody for 40 minutes in a dark room. The retinal sections were rinsed with PBS and were mounted with Vectorshield (Vector Laboratories, Burlingame, CA). A Zeiss LSM 700 confocal microscope system was used to observe fluorescence labeling in the retinal sections. Single scanning and multiple scanning along Z-direction were applied on the sections with the Zen software. The images were collected and processed with the software.

### Ca^2+^ imaging

Isolated retinal cells seeded on lectin-coated cover slips were incubated for 20-25 minutes in the cell-permeable calcium sensitive Fluo-4 AM dye (3μM in ringer solution). The cover slip was washed in Ringer’s solution before being placed in the recording chamber. Cells were excited at 480nm with the emission collected at 520nm. A Rolera-MGi Plus camera (Q-imaging) was used to view and collect fluorescence signals. Frame images were taken every 3 seconds with cells exposed for 50 milliseconds at each interval. A Lambda 10-2 (Sutter Instrument Co) controlled by IP Lab 4.0 software was used to open and close the filter shutter at each interval. All cell preparation and experimentation was done at room temperature in complete darkness.

The third-order neurons were identified by their morphology. Usually, dissociated ganglion cells appeared to have a long axon process extending from the cell somas; the dissociated amacrine cells have larger somas than that of bipolar cells. However, with the morphological profiles, we could not rule out the possibility that some bipolar and horizontal cells might be mistakenly counted in our experiments since most of dissociated cells lost their clear morphology.

### Data analysis

Electrophysiological data was obtained using HEKA Patchmaster software and analyzed using Igor Pro Software. Ca^2+^ imaging data was measured using IP Lab 4.0 software. In these experiments individual frames from a single experiment were combined into sequences before specific regions of interest were selected. For each cell in each sequence that showed a response the intensity value was normalized by first subtracting the baseline value before dividing by the maximum intensity for that cell. The normalized values for each experiment set were then combined to produce a mean value trace. Mean, Standard Error and paired t-test values were all calculated using Microsoft Office 2007.

### Chemicals

Taurine, Glycine, L-Glutamic acid, Lectin from *Triticum vulgaris*, Picrotoxin, Potassium D-gluconate, K-gluconate, BAPTA, EGTA, Adenosine 5’-Triphosphate (ATP), Phosphocreatine, Creatine Phosphokinase Type I, CoCl_2_, NaCl and Dextrose were purchased from Sigma-Aldrich Inc (St Louis, MO). L-Cysteine, EDTA, HEPES, MgCl_2_ and KCl were purchased from J.T. Baker (Phillipsburg, NJ). CaCl_2_ was purchased from Fisher Scientific (Fair Lawn, NJ). Papain and Deoxyribonuclease I were purchased from Worthington Biochemical (Lakewood, NJ). Kainic Acid, (S)-AMPA, NMDA, SR 95531, Bicuculline, TPMPA, CGP 55845, GF 10920X, H-7, H-89, KN-62, PKI 14-22 amide, Thapsigargin, Ruthenium Red, CNQX and L-AP7 were all purchased from Tocris Bioscience (Ellisville, MO). Fluo-4AM was purchased from Invitrogen Corporation (Carlsbad, CA).

## Results

### Taurine-containing neurons in the amphibian retina

**Figure 1:**
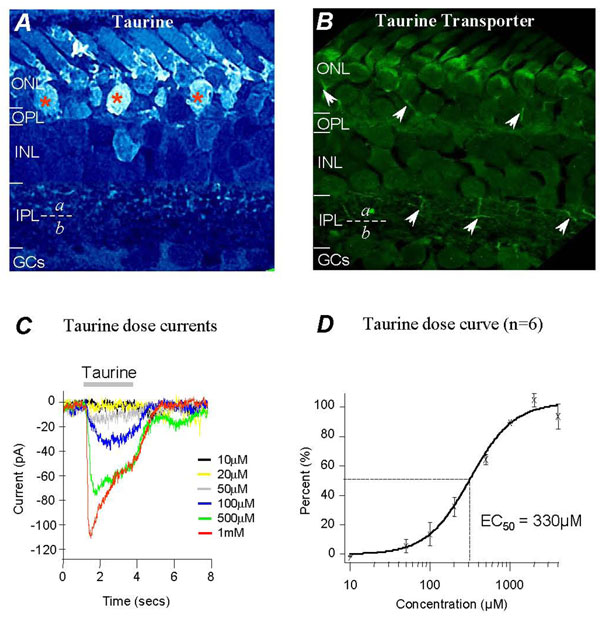
**Taurinergic neurons and taurine–dose response in salamander retina.** Immunoreactive pattern shows that taurine labeling is present in rods, a few cones and displaced Off-bipolar cells (asterisks) in the ONL. It is also present in cell somas in the INL and axon terminals in the sublamina of the IPL (A); anti-taurine transporter labels rod axons and Off-bipolar cell axons in the sublamina a of the IPL (B). Whole-cell recording of taurine dose response currents from an isolated ganglion cell (C). Taurine dose-response curve and EC_50_ value were obtained from the third-order neurons.

The taurine-positive neurons in tiger salamander retina were labeled by the specific antibody of taurine in frozen vertical sections. Figure 1A shows the immunocytochemical results, indicating that the anti-taurine labeling was present in the somas and processes of rods and a few cones, as well as in the displaced bipolar cells located in the outer nuclear layer (see asterisks). Displaced bipolar cells in the outer nuclear layer (ONL) of salamander retina have been characterized as Off-type bipolar cells in a previous study [56]. The anti-taurine labeling was also present in the bipolar cell somas in the inner nuclear layer (INL) and the processes at the distal half of the inner plexiform layer (IPL). In contrast, no taurine antibody labeling was observed in the proximal half of the IPL. It is known that the axon terminals of On- and Off-bipolar cells in salamander retina are located separately at the proximal and distal half of the IPL respectively, therefore the taurine-positive labeling in the distal IPL indicates that Off-bipolar terminals may contain taurine. The taurine antibody-labeling results indicated that both amacrine and ganglion cell somas were taurine negative in salamander retina.

To further characterize taurinergic neurons, a specific taurine transporter antibody was used. Figure 1B shows transporter antibody labeled photoreceptor axones and some processes at the distal sublamina of the IPL in retinal sections (see arrows). The result of taurine transporter labeling was consistent with taurine labeling further indicating that Off-bipolar cells might be taurinergic neurons releasing taurine. As Off-bipolar cells are also glutamatergic neurons, our results suggest that these neurons might release both glutamate and taurine. Therefore, amacrine and ganglion cells as the third-order neurons receive juxtaposed inputs of glutamate and taurine in the IPL.

### Taurine dose responses in isolated third-order neurons

To study the sensitivity of taurine, taurine dose-dependent currents were studied in dissociated third-order neurons in whole-cell voltage-clamp mode. The cells were identified by their electrical properties with voltage-dependent Na^+^ currents in multiple depolarizing voltage steps. Photoreceptors, bipolar cells and horizontal cells were lacking Na^+^ currents, enabling the third-order neurons in isolated cell preparations to be distinguished. Third-order neurons can be further characterized as Na^+^ currents are much smaller in amacrine cells compared to those in ganglion cells. The cells were voltage-clamped at -70mV, near the resting potential of the cells. Inward currents were recorded from the cells elicited with taurine concentrations of 10µM, 20µM, 50µM, 100µM, 500µM, 1mM. 2mM and 4mM puffed locally on the neurons. Figure 1C shows typical taurine currents elicited by various concentrations recorded from ganglion cells. The amplitudes of taurine currents were measured and plotted as a dose response relationship curve (Fig. 1D), showing that 10µM taurine elicited a negligible current with maximum currents elicited by 2mM taurine. A taurine concentration of 330µM elicited a half maximum response (EC_50_) determined from the dose response relationship curve (see dot-lines, Fig. 1D). Taurine dose response currents also indicate that concentrations of taurine below the EC_50_ value elicit sustained inward currents with limited desensitization. Transient currents with fast desensitization were observed when the concentration was higher than the EC_50_, as shown in figure 1C.

### Taurine suppressed glutamate-induced [Ca^2+^]_i_ in the third-order neurons

**Figure 2:**
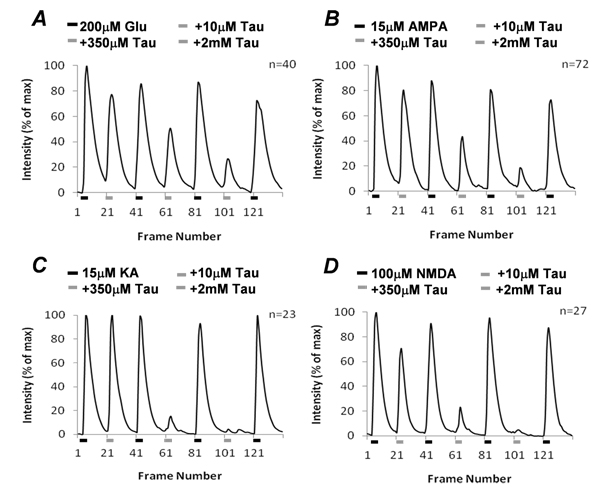
**Statistical results of taurine regulation of glutamate-induce [Ca^2+^]_i_ in isolated third-order neurons.** Taurine suppresses the [Ca^2+^]_i_ in the third-order neurons in a dose- dependent manner (A). Suppressive effects of taurine on AMPA-, kainate- and NMDA-induced [Ca^2+^]_i_ in the neurons (B, C and D).

The effect of taurine on glutamate response was studied in the third-order neurons using Ca^2+^ imaging. The isolated cells were loaded with a membrane permeable Ca^2+^ indicator Fluo-4-AM. The third-order neurons were identified by their morphology (see the Methods section). Application of 200µM glutamate evoked a large [Ca^2+^]_i_ increase from the third-order neurons. When taurine was co-applied with glutamate in 10µM, 350µM and 2mM, the minimum, EC_50_ and maximum concentrations respectively, the [Ca^2+^]_i_ was suppressed in a dose-dependent manner. Figure 2A shows the [Ca^2+^]_i_ changes in the third-order neurons activated by glutamate and glutamate with varying concentrations of taurine. On average, 10µM taurine reduced 23% of glutamate-elicited free [Ca^2+^]_i_ levels, with the EC_50_ and maximum concentration of taurine blocking 40% and 67% of the glutamate-elicited free [Ca^2+^]_i_ respectively in the third-order neurons (n=40). This indicates that the effect of taurine effectively suppressed glutamate-elicited [Ca^2+^]_i_ in the neurons.

As glutamate activates kainate-, AMPA- and NMDA-sensitive ionotropic receptors, we used the receptor agonists to separate activity at each single receptor subtype. Previous Ca^2+^ imaging studies indicate that low concentrations of kainate and AMPA specifically activate their own receptors that are highly permeable to Ca^2+^ in the third-order neurons [17]. Kainate and AMPA at concentrations of 15µM increased [Ca^2+^]_i_ in the neurons (Fig. 2B &2C). 10µM taurine reduced AMPA-induced [Ca^2+^]_i_ to 80% of the control (n=72), but had no significant effect on kainate-induced [Ca^2+^]_i_ with no reduction in the level of [Ca^2+^]_i_ (n=23). This indicates that the low concentration of taurine selectively suppressed AMPA-sensitive receptor response in the neurons. Higher taurine concentrations gradually suppressed AMPA-induced [Ca^2+^]_i_, but acutely reduced kainate-induced [Ca^2+^]_i_ in the neurons (Fig. 2B and 2C). Kainate-induced [Ca^2+^]_i_, but not AMPA-induced [Ca^2+^]_i_, was completely blocked by 2mM taurine. Since AMPA receptors, but not kainate receptors, are synaptic in the third-order neurons [18], the different effects of taurine on AMPA and kainate responses suggest that taurine might have alternate functions on synaptic and non-synaptic glu tamate receptors. The effect of taurine on NMDA receptors was also examined in the third-order neurons. On average, 10µM and 350µM taurine suppressed 29% and 77% of NMDA-induced [Ca^2+^]_i_ in the neurons, respectively (n=27). NMDA-induced [Ca^2+^]_i_ was completely blocked by 2mM taurine (Fig. 2D).

Taken together, these results indicate that taurine at higher concentrations from 350µM to 2mM could effectively block Ca^2+^-permeable kainate and NMDA receptors but have a lesser effect on Ca^2+^ -permeable AMPA receptors.

### The effect of taurine was sensitive to strychnine and picrotoxin, but not GABA receptor antagonists

**Figure 3:**
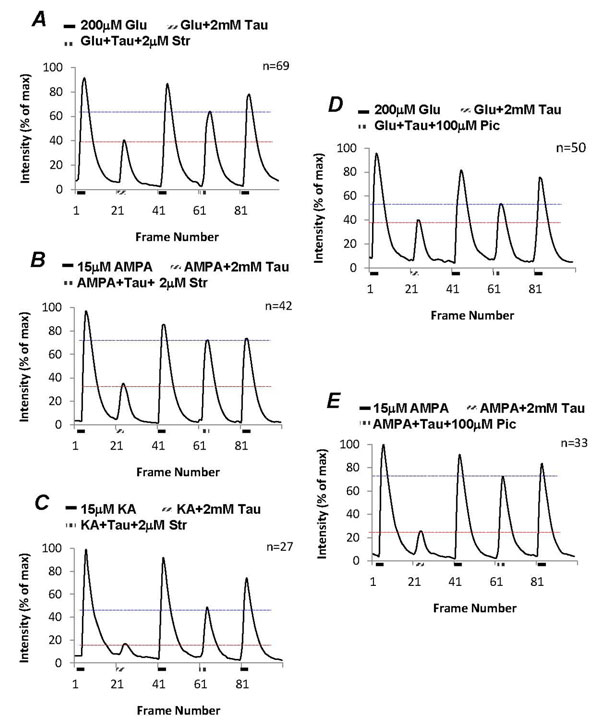
**Pharmacological study of taurine response in isolated third-order neurons.** Strychnine (2µM) partially reverses the suppressive effects of taurine on glutamate-, AMPA- and kainate-induced [Ca^2+^]_i_ from the third-order neurons (A, B and C). Picrotoxin (100µM) had similar effects as strychnine partially reversing the effects of taurine on glutamate- and AMPA-induced [Ca^2+^]_i_ (D and E).

As the third-order neurons possess both glycine and GABA receptors, the effect of taurine on suppression of glutamate-induced [Ca^2+^]_i_ was examined with the addition of various glycine and GABA receptors antagonists. Figure 3A shows that 2mM taurine suppressed glutamate-induced [Ca^2+^]_i_ by 55% (t=17.21, df=136, p<0.0001), which was significantly reversed by 2µM strychnine to 75% of the control (t=6.44, df=136, p<0.0001). The remaining strychnine-insensitive effect indicates that taurine might activate different types of receptors. The effect of taurine on AMPA- and kainate-induced [Ca^2+^]_i_ in the third-order neurons was also tested. In the control, 2mM taurine significantly suppressed both 15µM AMPA- by 65% (t=15.78, df=82, p<0.0001) and 15µM kainate-induced [Ca^2+^]_i_ by 85% (t=19.65, df=52, p<0.0001) in the third-order neurons, as shown in figure 3B and 3C. Though strychnine appeared to be more effective on blocking the effect of taurine on AMPA-induced [Ca^2+^]_i_ compared to kainate-induced [Ca^2+^]_i_, the reversal was significant for both AMPA (from 35% to 85%; t=9.4488, df=82, p<0.0001) and kainate (from 15% to 60%; t= 5.52, df=52, p<0.0001). These results suggest that the action of taurine on glutamate receptors could therefore be mediated by strychnine-sensitive receptors.

The effect of strychnine could be duplicated by 100µM picrotoxin, a non-selective blocker for Cl^-^ permeable receptors, including both glycine and GABA receptors. On average, picrotoxin significantly reversed the effect of 2mM taurine on glutamate-induced [Ca^2+^]_i_ from 55% to 30% (t=3.93, df=96, p = 0.0002), showing in figure 3D. Picrotoxin was more effective in blocking the taurine effect on AMPA-induced [Ca^2+^]_i_. Figure 3E shows that in control taurine suppressed about 70% of AMPA-induced response; with picrotoxin taurine having 20% suppression on the AMPA-induced response (t=12.18, df=64, p<0.0001). This indicates that the effect of taurine on glutamate-induced [Ca^2+^]_i_ is active through a Cl^-^ permeable receptor blocked by picrotoxin.

**Figure 4:**
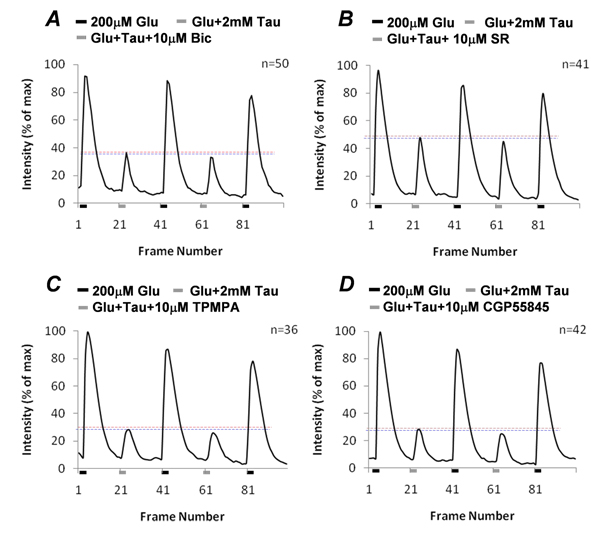
**GABA receptor antagonists had no effect on taurine-produced effects.** Neither GABA_A_ receptor antagonists, bicuculline (10µM) and SR95531 (10µM) (A and B), nor GABA_C_ and GABA_B_ receptor antagonists, TPMPA (50µM) and CGP55845 (10µM; C and D) block taurine suppression on glutamate-induced [Ca^2+^]_i_ .

The effect of picrotoxin and strychnine was not replicated by GABA receptor antagonists that had no significant effect on taurine suppression of glutamate-induced [Ca^2+^]_i_ . Figure 4A and 4B show that both GABA_A_ receptor antagonists, 10µM bicuculline (t=0.89, df=98, p=0.375) and 20µM SR95531 (t=0.59, df=80, p=0.5642) had no significant effect on reversing taurine-produced suppression on glutamate-induced [Ca^2+^]_i_ . In addition the GABA_C_ receptor antagonist 50µM TPMPA (t=0.26, df=70, p=0.7913) and the GABA_B_ receptor antagonist 10µM CGP55845 (t=0.77, df=82, p=0.4422) were also unable to reverse taurine-produced suppression (Fig. 4C and 4D). These results clearly show that the effect of taurine was unrelated to all GABA receptor subtypes.

### Taurine suppressed glutamate-induced Ca^2+^ influx through both glutamate receptors and voltage-gated Ca^2+^ channels

**Figure 5:**
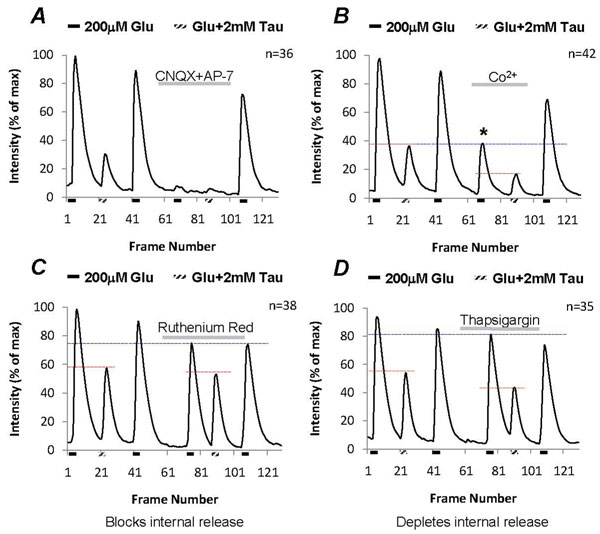
**Glutamate induces Ca^2+^ influx via both glutamate receptors and voltage-gated Ca^2+^ channels in third-order neurons.** (A) Glutamate-induced [Ca^2+^]_i_ is completely blocked by ionotropic glutamate receptor antagonists, CNQX (50µM) and AP-7(40µM). (B) Co^2+^ blocks voltage-gated Ca^2+^ channels, which reduces 55% of glutamate-induced [Ca^2+^]_i_ in the neurons (see asterisk); with Co^2+^ taurine still suppresses glutamate-induced [Ca^2+^]_i_. (C) Ruthenium red (1µM), a rynadine receptor inhibitor, reduces 15% of glutamate-induced [Ca^2+^]_i_; with ruthenium red, taurine has 35% suppression on glutamate-induced [Ca^2+^]_i_ in the neurons. (D) Blocking Ca^2+^ uptake pumps in the internal organelles with thapsigargin (1µM) had a limited effect on taurine regulation of [Ca^2+^]_i_.

Glutamate elicited [Ca^2+^]_i_ in the third-order neurons could be the result of activation of both metabotropic and ionotropic receptors. Therefore to separate their individual effects, CNQX (6-cyano-7-nitroquinoxaline-2,3-dione) and AP-7 (D,L-2-amino-*7*-phosphonoheptanoic acid) were used to block all ionotropic glutamate receptors. The glutamate-induced [Ca^2+^]_i_ was completely blocked by the presence of these ionotropic glutamate receptor antagonists, and in addition when 2mM taurine was co-applied it had no effect on [Ca^2+^]_i_ in the neurons (Fig. 5A), suggesting that activation of ionotropic glutamate receptors causes a raise of [Ca^2+^]_i_ in the third-order neurons.

Glutamate depolarizes the third-order neurons leading to activation of voltage-gated Ca^2+^ channels, therefore glutamate-induced [Ca^2+^]_i_ could be the result of Ca^2+^ influx through both glutamate receptors and voltage-gated Ca^2+^ channels. To separate [Ca^2+^]_i_ influx via glutamate receptors from voltage-dependent Ca^2+^ channels, 1mM cobalt was used to block voltage-gated Ca^2+^ channels. On average, cobalt reduced about 55% of the glutamate-induced [Ca^2+^]_i_ (n=42, Fig. 5B, see asterisk). The remaining cobalt-insensitive [Ca^2+^]_i_ was further suppressed by 2mM taurine, indicating that taurine suppressed Ca^2+^ influx through ionotropic glutamate receptors.

### Glutamate had a limited effect on triggering internal Ca^2+^ release in isolated neurons

In many cases, Ca^2+^ influx could stimulate Ca^2+^ release from the intracellular Ca^2+^ stores. To assess the contribution of Ca^2+^ release from internal stores, 1.5µM ruthenium red was used to block ryanodine receptors in the intracellular organelles, which blocked Ca^2+^-sensitive release from internal stores in the third-order neurons. The effects of taurine on glutamate-induced Ca^2+^ increase were examined in the control and with ruthenium red. In the control, taurine suppressed 55% of glutamate-induced [Ca^2+^]_i_ in the neurons, ruthenium red approximately reduced 15% of glutamate-induced [Ca^2+^]_i_ (Fig. 5C), glutamate had a limited effect on triggering ryanodine receptor -sensitive internal Ca^2+^ release. With the blockage of Ca^2+^ release from internal stores, taurine suppressed 35% of the total glutamate-induced [Ca^2+^]_i_. Since the taurine suppression was reduced in the presence of ruthenium red, this indicates that the effect of taurine might reduce glutamate-induced internal Ca^2+^ release via ryanodine receptors.

Another approach was to use thapsigargin to block the Ca^2+^-uptake pump in intracellular Ca^2+^ release organelles resulting in depletion of internal Ca^2+^ release stores. The same experimental protocol was applied for the control and with the presence of 1.5µM thapsigargin. Figure 5D demonstrates that thapsigargin pre-perfusion only caused 5% reduction of glutamate-induced [Ca^2+^]_i_ compared to the control. Depleting internal Ca^2+^ release stores had no significant effect on taurine suppression of glutamate-induced [Ca^2+^]_i_ . With thapsigargin, taurine still reduced approximately 45% of glutamate-induced [Ca^2+^]_i_ similar to the effect of taurine in control.

The above results confirm that Ca^2+^ influx stimulated internal Ca^2+^ release played a minor role in glutamate-induced [Ca^2+^]_i,_ therefore the major cause of glutamate-induced [Ca^2+^]_i_ was Ca^2+^ influx through receptors in isolated neurons. Possibly, the effect of taurine was via regulation of Ca^2+^ permeability of glutamate receptors and voltage-gated Ca^2+^ channels.

### Taurine regulation of glutamate-induced [Ca^2+^]_i_ was sensitive to CaMKII and PKA inhibitors

**Figure 6:**
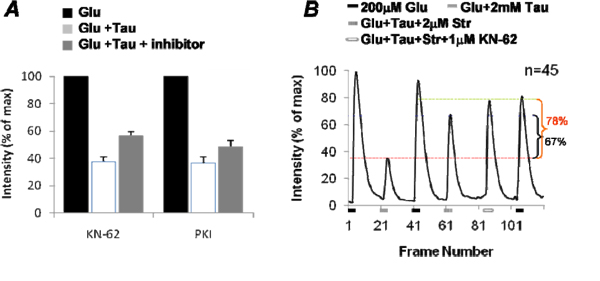
**Pharmacological evidence of CaMKII and PKA involved in taurine regulation of glutamate-induced [Ca^2+^]_i_ .** (A) Statistical results of the selective cell-permeable CaM kinase II inhibitor KN-62 and protein kinase A inhibitor PKI (14-22) amide (both 1µM) on taurine regulation of glutamate-induced [Ca^2+^]_i_ in isolated third-order neurons. (B) CaMKII involved in the strychnine-insensitive taurine regulation on glutamate response.

If intracellular signal transduction pathways are involved in taurine regulation, then their inhibitors should reverse the suppressive potential of taurine. Several intracellular protein inhibitors were used to block taurine produced effect and among these inhibitors the selective cell-permeable CaMKII inhibitor KN-62 and protein kinase A inhibitor PKI (14-22) amide (both 1µM) could partially reverse the taurine-produced suppressive effect on glutamate responses. Figure 6A shows the statistics of the reductions of glutamate-induced [Ca^2+^]_i_ by taurine in control and in the presence of KN-62 or PKI (14-22). In the control, taurine suppressed glutamate-induced [Ca^2+^]_i._ by 62%+3% (n=40). Application of KN-62 had no effect on glutamate-induced [Ca^2+^]_i_., however with KN-62, taurine suppression was reversed significantly to 47%+3% (n=40, t=4.44, df=78, p<0.0001). In the case of PKI (14-22) amide, taurine suppressed glutamate-induced [Ca^2+^]_i_ by 63%+5% (n=38) in the control, which was reduced to 52%+4% (t=1.99, df=74, p=0.0498) with blockade of PKA. This indicates that both CaMKII and PKA are the intracellular proteins involved in the taurine signaling transduction.

As shown in figure 3A the taurine suppression of glutamate-induced [Ca^2+^]_i_ was via both the strychnine-sensitive and -insensitive receptors, CaMKII and PKA and could involve the downstream pathways of these taurine-activated receptors. To specify which type of taurine-activated receptors triggers the CaMKII-sensitive regulation in glutamate-induced [Ca^2+^]_I_, we performed the experiments with strychnine to block strychnine-sensitive taurine receptors, isolating strychnine-insensitive taurine receptor response. Figure 6B is the statistical results from 48 cells, showing that 2µM strychnine blocked 67% of taurine-caused suppression on glutamate-induced [Ca^2+^]_i_. With strychnine, KN-62 further blocked about 11% of the taurine suppression on glutamate-induced [Ca^2+^]_i_ (t=3.60, df=88, p=0.0005), indicating that CaMKII mediates strychnine-insensitive taurine regulation in the third-order neurons.

### Evidence of Ca^2+^ -dependent regulation of taurine response

**Figure 7:**
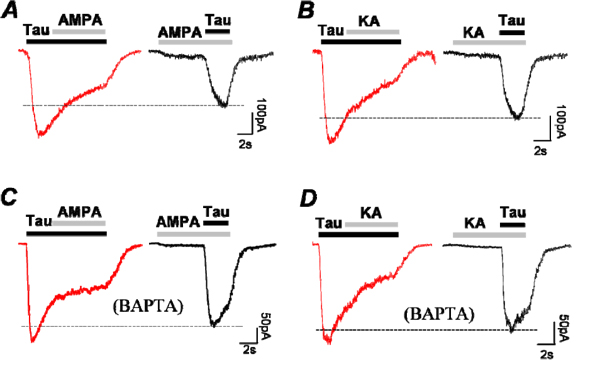
**Taurine-elicited currents are suppressed by AMPA and kainate in isolated third-order neurons.** (A and B) 350µM taurine elicits currents that are suppressed by co-application of either 15µM AMPA or kainate; both amplitudes and kinetics of the taurine currents are altered in the presence of AMPA or kainate. (C and D) the same experiments repeated with intracellular application of 10mM BAPTA.

We found that while taurine regulates glutamate response, Ca^2+^ influx via glutamate receptors also regulates taurine response. This was studied in a whole-cell voltage-clamp recording mode. Taurine-elicited currents were measured in the presence and absence of glutamate receptor agonists. As the results in figure 2 show that activation of AMPA and kainate receptors could effectively raise [Ca^2+^] levels, both 15µM AMPA and kainate were used to elevate [Ca^2+^]_i_ levels in the neurons. A taurine dose of 350µM was applied before the application of AMPA or kainate. Taurine elicited a large inward current with fast desensitization at a holding voltage -70mV, and adding AMPA or kainate had no significant change on the substandard taurine currents (the left panels, Fig. 7A &7B). After pre-application of AMPA and kainate however, the amplitudes of taurine currents were reduced in the same cells, and the kinetics of the onset currents was much slower when compared to the control (the right panels, Fig. 7A &7B). Although 15µM AMPA and kainate alone could elicit large [Ca^2+^]_i_ increases (see Fig. 2B and 2C), the currents elicited by these agonists were small at -70mV. Therefore, the effect of AMPA and kainate on taurine-elicited currents might be mainly caused by an increase in [Ca^2+^]_i_ levels, rather than conductance change of the neurons.

To further identify that increasing [Ca^2+^]_i_ is the key for altering taurine response, a fast Ca^2+^ chelator BAPTA was applied to buffer [Ca^2+^]_i_ in the micro-domain regions under the plasma membranes of the cells. BAPTA (10mM) was applied into the cytosol through recording electrodes during whole-cell recording. It was determined that BAPTA reduced the effects of AMPA and kainate on taurine-elicited currents (6 cells out of 8 recordings, Fig. 7C &7D). With BAPTA buffer, both kinetics and amplitudes of taurine currents increased suggesting that intracellular free Ca^2+^ is a key element for regulation of taurine currents by activation of glutamate receptors in the third-order neurons.

**Figure 8:**
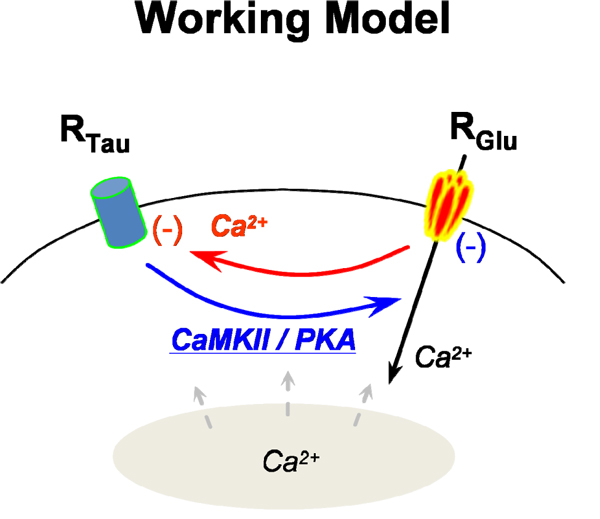
Potential intracellular pathways for reciprocal regulations between taurine-sensitive receptor and glutamate receptor.

Taken together, our study indicates that taurine and glutamate reciprocally inhibit each other in the third-order neurons. Figure 8 depicts the intracellular regulation between taurine and glutamate, which summarizes our findings. Taurine inhibits glutamate-induced [Ca^2+^]_i_ via a CaMKII pathway. Meanwhile rapid a increase of intracellular free Ca^2+^ by activation of AMPA and kainate receptors in turn negatively controls taurine response in the retinal third-order neurons. However, internal Ca^2+^ release from the cellular organelles seemed to be less effective in the intracellular regulation pathways between taurine and glutamate in isolated third-order neurons.

## Discussion

### Taurine and glutamate might be released from the same cells

This study shows with specific antibody labeling that the amphibian retina contains both taurine and taurine transporter in photoreceptors and Off-type bipolar cells. The presence of taurine in photoreceptors is consistent with previous studies from mammalian and non-mammalian animals [11,19]. Taurine has been also reported to present On-bipolar cells, amacrine and ganglion cells in various species during development. In rat retina taurine transporter has been observed in photoreceptors, bipolar cells and Müller cells [7]. All these suggest that in general taurine and glutamate are likely present in the same neurons and possibly are released from the same cells in retinas. However, no evidence suggests that glutamate and taurine would co-release from same vesicles.

It is unclear how taurine releases in the CNS. In retinas both light stimulation and high potassium depolarization increase extracellular levels of taurine, glutamate and GABA [9,19-21]. This suggests that the mechanism for taurine release might be similar to Ca^2+^-dependent release of glutamate and GABA. However, other mechanisms might exist for taurine synaptic release in neurons, for instance, taurine release from glutamatergic cerebellar granule cells is Ca^2+^-independent [22]. Hyposmotic stimulation can also result in taurine release [23-25]. These could be potential mechanisms for taurine release in photoreceptors and Off-bipolar cells in amphibian retina. Since taurine and the taurine transporter are not present in Müller cells in the amphibian retina, it is likely that taurine is released solely from neurons in this species. Our study further demonstrates that the synergistic release of taurine and glutamate provides reciprocal regulatory effects in the postsynaptic neurons.

### Pharmacology of taurine-produced effect in retinal third-order neurons

Taurine has been shown to activate GABA_A_ receptors in rat hippocampal CA1 area [26] and interact with GABA_B_ binding sites in mouse brain [27]. The taurine inhibitory effect is also sensitive to glycine receptor antagonist strychnine, suggesting that taurine could be acting through both GABA and glycine receptors [28]. Studies also suggest that taurine action on GABA and glycine receptors is usually dependent on several factors, such as concentration, specific region of the CNS and animal age [29]. In this study we find that the 2mM taurine-produced effect was partially blocked by strychnine and picrotoxin, but not by any GABA receptor antagonists. This finding is consistent with observations in isolated frog spinal cord [30]. If taurine has a lower potency to GABA receptors than to glycine receptors in the amphibian third-order neurons, the taurine concentration used in this study might be too low to activate GABA receptors since previous studies show that in the 10µM and 1mM range taurine activates glycine receptors while higher concentrations of taurine (1mM-10mM) activate GABA_A_ receptors in brain cells [31-34]. The highest concentration of taurine we used is on the low end of the GABA receptor activation but based on the saturating concentration from whole-cell recording (Fig. 1D). Molecular studies reveal that the sensitivity of GABA receptors to taurine is also dependent on receptor subunit compositions. Recombinant GABA_A_ receptor studies indicate that taurine is more potent to GABA_A_ receptors that consist of α,β,δ subunits than those with α,β,γ subunit combinations [3,35]. Possibly, GABA_A_ receptors in amphibian third-order neurons likely lack δ subunits, which results in a low sensitivity of those receptors to taurine. By this nature, we postulate that taurine release from Off-bipolar cells may not significantly interfere with GABAergic inputs in the third-order neurons. Taurine may be acting on third-order neurons primarily through either glycine receptor or a further unidentified taurine receptor [36] that is sensitive to strychnine and picrotoxin. So far, it is difficult to differentiate taurine-sensitive receptors from glycine and GABA receptors or to study strychnine-insensitive taurine response due to lack of pharmacological tools. Also, there is no molecular evidence that taurine has its own receptors.

### Potential mechanisms involved in taurine regulation	

Several findings are present in this study: 1) taurine regulates Ca^2+^ permeable glutamate receptors via CaMKII- and PKA-dependent intracellular pathways indicating that the regulation is not due to blockage of glutamate receptors by taurine. The result is in agreement with the previous studies that taurine does not bind to any type of glutamate receptor directly, including the glycine binding site in NMDA receptor [43]. 2) Likely taurine regulates glutamate induced Ca^2+^ influx by either hyperpolarizing the cell therefore reducing glutamate excitation or by decreasing Ca^2+^ permeability of glutamate receptors via intracellular second messengers. Indeed, taurine regulated glutamate response is dependent on both strychnine-sensitive and -insensitive manners in the third-order neurons (Fig. 3). The strychnine-sensitive effect of taurine depicts an ionotropic effect that suppresses glutamate excitation by hyperpolarizing the cells to the Cl^-^ reversal potential that is more negative than the resting membrane potential in the third-order neurons, whereas the evidence of strychnine-insensitive effect via a CaMKII-mediated intracellular pathway (Fig. 6) indicates that a metabotropic response of taurine exists in the retinal third-order neurons. 3) Taurine regulations of L-, N and P/Q-type voltage-gated Ca^2+^ channels, as well as Ca^2+^-permeable NMDA receptors in cultured brain neurons, have been reported previously [45]. As consistent with the previous studies, our results indicate that taurine reduces [Ca^2+^]_i_ accumulations in the third-order neurons via not only Ca^2+^ permeable glutamate receptors, but voltage-gated Ca^2+^ channels since Co^2+^ reduces a large amount [Ca^2+^]_i_ induced by glutamate (Fig. 5B). Both NMDA and non-NMDA glutamate receptors are Ca^2+^ permeable in the amphibian third-order neurons, which has been identified in previous studies [17,44]. We demonstrated that taurine regulate both Ca^2+^-permeable glutamate receptors in the neurons. 4) Ca^2+^ is the second messenger for modulation of taurine-sensitive receptors in the neurons. Glutamate-induced accumulation of [Ca^2+^]_i_ could activate both Ca^2+^/calmodulin and PKA through a Ca^2+^/calmodulin-dependent mechanism. Also Ca^2+^-dependent PKC might be the mechanism for regulation of taurine-sensitive receptors. The detail mechanism for Ca^2+^ -related regulation of taurine-sensitive receptors needs to be further investigated.

The selective inhibitor of CaMKII has a significant effect on taurine regulation and the PKA inhibitor seems also to block taurine regulation to a smaller extent. It is unclear whether these two cellular proteins work in parallel or series pathways. Yao and Wu report that Ca^2+^ directly binds to Ca^2+^/calmodulin to activate CaMKII, then Ca^2+^/calmodulin dependent activation of adenylyl cyclase stimulates cAMP synthesis that in turn activates PKA [37]. Since CaMKII can activate PKA, the results with PKI (14-22) experiments suggest PKA might be a down-stream protein of CaMKII pathway in the taurine regulatory pathway. The CaMKII inhibitor KN-62 was more effective at suppressing the taurine effect possibly because it is further upstream in the signal transduction pathway. We also speculate whether the CaMKII and PKA inhibitors directly suppress taurine-sensitive receptors. This scenario could also cause a reduction of the taurine suppression on glutamate-induced [Ca^2+^]_i_. Previous studies have indicated that CaMKII modulates glycine and GABA_A_ receptor activations by regulation of phosphorylation states [40-42]. Ca^2+^-dependent regulations are involved in up-regulation of taurine uptake [38] and taurine responses to osmotic changes in neurons and astrocytes [39]. Therefore, it is possible that CaMKII and PKA directly regulate taurine-sensitive receptors in the retinal neurons. CaMKII-dependent regulations seem to be widely presented in taurine regulations in the CNS. In spite of CaMKII-dependent pathway, other pathways might be also involved in taurine regulation, such as glutamate-induced PKC that have also been shown to be down-regulated by taurine [43]. Our study does not exclude other pathways involved in taurine regulation of glutamate response.

In general, activation of glutamate receptors leads to an extracellular Ca^2+^ influx as well as mobilization of intracellular Ca^2+^ stores [46]. However, in an isolated cell preparation, we observed that internal Ca^2+^ release was much less sensitive to glutamate application. This could be due to a depletion of internal Ca^2+^ stores caused by the process of enzyme-treatment and cell dissociation. In these conditions, activation of metabotropic glutamate receptors had very limited effect on stimulating internal Ca^2+^ release since the release stores were under depleted conditions. Therefore, our study did not rule out the possibility that taurine regulation of metabotropic glutamate receptors in the third-order neurons may also occur.

### Possible functions of taurine in glutamate transmission and neuroprotection

Taurine concentration in retinal tissues is extremely high. Studies in goldfish retina show that the concentration of taurine is almost 20 times higher than that of glutamate and as much as 25 times higher than GABA [9]. Insufficient taurine in the embryonic stage of photoreceptor development has been shown to lead to blindness in newborns [25,50,51]. Supplementation of taurine in the diet can reverse the damage caused in retinal development by taurine deficiency [52]. Clearly, taurine is important for retinal development and maturation.

Early studies have shown that taurine suppresses light-evoked responses in Off-bipolar cells in mudpuppy retinas as well as light-evoked off responses in transient ganglion cells in frog retinas [47-49]. Our results reinforce the previous findings that taurine is a neurotransmitter/modulator released from Off-bipolar cells to directly activate amacrine and ganglion cells. The function of taurine regulation of Ca^2+^ permeability in glutamate receptors might relate to the control of glutamate signals in the Off-pathway. In fact, taurine and glutamate are reciprocally inhibiting each other. This suggests that the regulation between glutamate and taurine may contribute to fine-tuning light signals in the third-order neurons.

Taurine has multiple functions in neuroprotection against glutamate toxicity in the central brain [45,51,53]. Taurine has been shown to increase mitochondrial function of buffering [Ca^2+^]_i_ to protect cerebellar granule cell damages in excitotoxicity [54]. Wu et al., (2009) have reported that taurine can effectively reduce Ca^2+^ overload via voltage-gated Ca^2+^ channels and NMDA glutamate receptors via intracellular calpain-dependent mechanisms [45]. This leads to anti-apoptosis in brain neurons. Indeed, taurine levels are increased after brain ischemia and spinal cord injury in which neurons are susceptible to the damage from glutamate toxicity [55]. This might indicate taurine as a natural neuroprotective chemical in the CNS. Taurine inhibits glutamate-induced [Ca^2+^]_i_ overloading might be the key function for neuroprotection of taurine against cell damage by excessive glutamate levels in pathological conditions. The results from our study suggest that taurine might have similar effects as in the central brain and spinal cord to protect retinal neurons form glutamate toxicity damage in disease.

## Competing interests

The authors declare that they have no competing interests.
